# Ginseng Stem-and-Leaf Saponins Mitigate Chlorpyrifos-Evoked Intestinal Toxicity In Vivo and In Vitro: Oxidative Stress, Inflammatory Response and Apoptosis

**DOI:** 10.3390/ijms242115968

**Published:** 2023-11-04

**Authors:** Silu Liu, Xiaoying Zhu, Hongyan Pei, Yan Zhao, Ying Zong, Weijia Chen, Zhongmei He, Rui Du

**Affiliations:** 1 College of Chinese Medicinal Materials, Jilin Agricultural University, Changchun 130118, China; liusilu0616@163.com (S.L.); m15886257839@163.com (X.Z.); phy19990505@163.com (H.P.); zhaoyan@jlau.edu.cn (Y.Z.); zongying7699@126.com (Y.Z.); chenweijia_jlau@163.com (W.C.); 2 Jilin Ginseng Academy, Changchun University of Chinese Medicine, Changchun 130118, China; 3 Key Laboratory of Animal Production, Product Quality and Security, Ministry of Education, Jilin Agricultural University, Changchun 130118, China

**Keywords:** OP, GSLS, CPF, oxidative stress, enterotoxicity, inflammatory response

## Abstract

In recent years, the phenomenon of acute poisoning and organ damage caused by organophosphorus pesticides (OPs) has been a frequent occurrence. Chlorpyrifos (CPF) is one of the most widely used organophosphorus pesticides. The main active components of ginseng stems and leaves are total ginseng stem-and-leaf saponins (GSLSs), which have various biological effects, including anti-inflammatory, antioxidant and anti-tumor activities. We speculate that these could have great potential in the treatment of severe diseases and the relief of organophosphorus-pesticide-induced side effects; however, their mechanism of action is still unknown. At present, our work aims to evaluate the effects of GSLSs on the antioxidation of CPF in vivo and in vitro and their potential pharmacological mechanisms. Mice treated with CPF (5 mg/kg) showed severe intestinal mucosal injury, an elevated diamine oxidase (DAO) index, the decreased expression of occlusive protein-1 (ZO-1) and occlusive protein, an impaired intestinal mucosal oxidation system and intestinal villi relaxation. In addition, chlorpyrifos exposure significantly increased the contents of the inflammatory factor TNF-α and the oxidative-stress-related indicators superoxide dismutase (SOD), catalase (CAT), glutathione SH (GSH), glutathione peroxidase (GSH-PX), reactive oxygen species (ROS) and total antioxidant capacity (T-AOC); elevated the level of lipid peroxide malondialdehyde (MDA); reversed the expression of Bax and caspase; and activated NF-κB-related proteins. Interestingly, GSLS supplementation at doses of 100 and 200 mg/kg significantly reversed these changes after treatment. Similar results were observed in cultured RAW264.7 cells. Using flow cytometry, Hoechst staining showed that GSLSs (30 μg/mL, 60 μg/mL) could improve the cell injury and apoptosis caused by CPF and reduce the accumulation of ROS in cells. In conclusion, GSLSs play a protective role against CPF-induced enterotoxicity by inhibiting NF-κB-mediated apoptosis and alleviating oxidative stress and inflammation.

## 1. Introduction

Organophosphorus pesticides (OPs) are widely used in agricultural and forestry production. They have the characteristics of high efficiency, a wide spectrum, low price and easy decomposition [1]; however, they are also toxic to humans and animals and can cause organophosphorus poisoning through different routes, such as through the skin [2], intestinal tract [3] or respiratory tract [4]. According to statistics, the death rate of patients with organophosphorus pesticide poisoning exceeds 10% every year [5]. The symptoms of patients with severe organophosphorus poisoning progress rapidly, with early complications such as respiratory failure, cardiac arrest, shock, gastrointestinal bleeding and multiple organ dysfunction syndrome [6,7,8]. Severe organophosphorus poisoning can result in multiple organ failure due to multiple organ function damage [9].

CPF (chlorpyrifos) is one of the most widely used OPs. With the aggravation of pesticide residues, the relevant test results showed that a large amount of CPF is often left in food, with the highest rate reaching 40% [10]. There is a balance between apoptosis and renewal in the intestinal tissues of healthy organisms [11]. However, CPF exposure may upset this balance, slowing the ability of cells to renew, enhancing intestinal barrier permeability and inducing enterotoxicity. In addition, in vivo and in vitro studies have shown that the potential mechanism of CPF-exposure-induced intestinal toxicity includes apoptosis [12], oxidative stress, inflammation and damage to the intestinal barrier [13].

Oxidative stress refers to a state of imbalance between oxidation and antioxidation in the body and manifests as the production of excessive reactive oxygen species (ROS) [14,15]. Oxidative stress usually causes serious oxidative damage to important components of cells and ultimately impairs cell viability [16,17]. ROS can be balanced by a complex antioxidant system to maintain redox homeostasis in cells [18]. The total antioxidant capacity (T-AOC), superoxide dismutase (SOD), catalase (CAT), glutathione SH (GSH) and glutathione peroxidase (GSH-Px) all belong to the antioxidant system [16]. Under physiological and pathological conditions, sources of reactive oxygen species (ROS) are abundant in organisms; irreversible damage may lead to the degradation and accumulation of damaged proteins in cells and eventually lead to organ damage in organisms [19]. The toxic environmental pollutant 4-tert-butylphenol decreased antioxidants (SOD, CAT, GSH and GSH-Px), increased oxidants (MDA (malondialdehyde) and ROS) and caused oxidative stress in common carp hepatocytes and led to increases in nuclear transcription factor-κB (NF-κB) and TNF-α and decreases in IκBα and inflammation in the gills of the common carp after exposure to the hazardous substance 4-octylphenol. Manganese toxicity downregulated antioxidants (SOD, CAT and GPx) and the tight-junction protein occludin, upregulated proinflammatory factors (NF-κB and TNF-α) and caused oxidative stress, inflammation and tight-junction dysfunction in common carp kidneys [20,21]. Li X et al. (2023) [22] measured oxidative stress indexes (SOD, GSH, T-AOC and MDA) and apoptosis-related genes (Bax, Bcl-2 and Caspase-3) and found that CPF-exposure-induced oxidative stress and apoptosis are common in carp gills. Wang et al. (2021) observed significant protein cleavage in HepG2 cells modeled with a mixture of dibenzonazole, cyclothrin and triazophosphorus. The mixture of pesticides significantly increased the ROS level, inhibited DNA synthesis, caused oxidative stress in cells, reversed the expression of inflammatory cytokines and induced apoptosis by destroying cell membranes to produce large amounts of lactate dehydrogenase [23].

The intestine is completely connected to the external environment. It is a standard external organ and the first physical barrier to resist external substances [24,25]. The intestinal epithelial barrier is primarily maintained by tight-junction proteins, which absorb nutrients in the diet and prevent pathogens and toxins from entering the body [26,27]. At the same time, these structures are susceptible to oxidative stress, as the pathological feature of redox imbalance is the breakdown of tight-junction complexes [28]. Therefore, oxidative stress is also considered to be an important indicator of intestinal damage [29]. Studies have shown that a variety of intestinal diseases and dysfunction can be attributed to the overproduction of ROS [30]. In addition, oxidative stress also reduces the height, surface area and volume of intestinal villi, ultimately affecting basic intestinal function. Thus, maintaining a balance between intestinal redox states is crucial.

Studies have confirmed that detoxification of the damage caused by OPs due to severe and oxidative stress can be achieved by supplementation with active biomolecules [3,31], medicinal plants and antioxidants. However, the current pharmaceutical detoxification measures for organophosphorus poisoning are still relatively limited, and the most common detoxification pathways are divided into two types: (1) antagonists, such as atropine [32], which antagonizes and eliminates muscarinic-like symptoms and central nervous system symptoms, excites the respiratory center and is a mandatory drug for detoxification treatment; (2) compounding agents, the most common of which are dephosphoridine and chlorophosphoridine.

However, these detoxification drugs are only symptomatic treatments, and all of them have drawbacks: atropine does not antagonize nicotine-like effects and cholinesterase reactivation [33], and in moderate and severe poisoning, it also needs to be combined with reactivators, and an overdose may lead to atropine poisoning [33]; organophosphorus and cholines are not solid in combination, the reactivation of certain organophosphorus pesticides is poor, and an overdose may lead to compounding agent poisoning [34], symptoms similar to organophosphorus poisoning [35] and significant side effects and obvious adverse reactions. Traditional Chinese medicine has been shown to have the advantages of unique efficacy, a remarkable therapeutic effect and minimal toxic side effects in the treatment of diseases.

Ginseng (Panax ginseng C.A. Meyer) is widely distributed in northeast China. Its functions include invigorating vitality, the spleen and the lungs; calming the mind; and improving the brain [36,37]. Ginseng stem-and-leaf saponins are the main active components of ginseng and have antioxidant [38], anti-inflammatory [39], anti-apoptotic [40] and protective effects on the nervous system. Ginsenoside Re (G-Re), which is abundant in GSLSs, is often used as a focus for studying the activity of chemical constituents with a wide range of pharmacological activities. In the present study, an intestinal injury model was established through exposure to CPF to evaluate the potential therapeutic mechanisms of the total saponins from ginseng stems and leaves. GSLSs have been demonstrated to be useful in the treatment of a wide range of disorders, including cardiovascular and cerebrovascular diseases [40]. Increasing evidence suggests that GSLSs play a key therapeutic role against cyclophosphamide-induced genotoxicity and apoptosis in mouse myeloid cells and peripheral lymphocytes [41]. However, the mechanism by which GSLSs attenuate CPF-induced intestinal toxicity in vivo and in vitro has not been reported.

## 2. Results

### 2.1. HPLC Analysis of Saponin Monomers in GSLS

**Figure 1:**
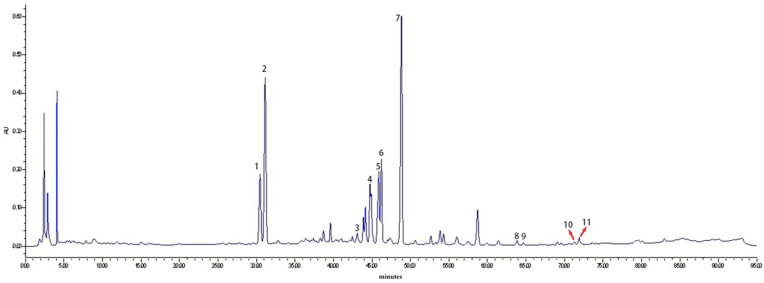
HPLC of total ginsenosides in stems and leaves of ginseng (GSLSs). These saponins mainly include ginsenoside Rg1, ginsenoside Re, ginsenoside Re, ginsenoside Rb1, ginsenoside Rc, ginsenoside Rb2, ginsenoside Rb3, ginsenoside Rd, 20(S)-ginsenoside Rg3, 20(R)-ginsenoside Rg3, ginsenoside Rk1, ginsenoside Rg5. Supplement:1-ginsenoside Rg1, 2-ginsenoside Re, 3-ginsenoside Rb1, 4-ginsenoside Rc, 5-ginenoside Rb2, 6-ginenoside Rb3, 7-ginsenoside Rd, 8-20 (S) ginsenoside Rg3, 9-20 (R)-Ginsenoside Rg3, 10-ginsenoside Rk1, 11-ginsenoside Rg5.

High-performance liquid chromatography (HPLC) is extensively utilized for the analysis of natural drugs. The HPLC chromatographic diagram of GSLSs in this study is shown in Figure 1, showing that it contained rich chemical components. The main compounds of GSLSs were further analyzed, and the quantitative determination results of crude saponins were as follows. Rg1: 7.04%; Re: 25.79%; Rb1: 3.42%; RC: 7.88%; Rb2: 9.94%; Rb3: 5.37%; Rd: 17.33%; (S)-Rg3: 1.97%; (R)-Rg3: 1.72%; Rk1: 1.47%; Rg5: 2.49%.

### 2.2. GSLSs Can Improve CPF-Induced Oxidative Stress and Enhance Antioxidant Activity

**Figure 2:**
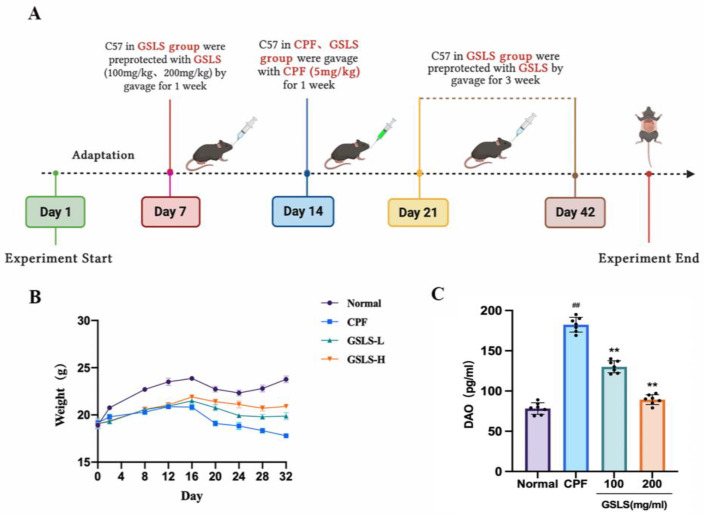
GSLSs improved CPF-induced intestinal oxidative stress damage. (**A**) Experimental protocol. (**B**) Effect of GSLSs on body weight changes in CPF-induced enterotoxicity mice. (**C**) DAO activity in intestinal tissue. All values are expressed as mean ± SD (*n* = 8); ^##^ *p* < 0.01 compared with the control group; ** *p* < 0.01 compared with the CPF group.

As shown in Figure 2B, the body weight of mice remained unaltered for the first 7 days after GSLS administration. Nevertheless, after CPF exposure, mice in all experimental groups experienced a gradual decrease in weight compared to the control group. However, the weight loss of mice in the GSLS-L group and the GSLS-H group appeared to be more moderate than that in the CPF group (*p* < 0.05).

DAO serves as a crucial indicator when assessing the intestinal barrier function, as shown in Figure 2C. Compared with the CPF group, the abnormal increase in DAO activity in intestinal tissue was significantly improved in the GSLS-L group and the GSLS-H group, indicating that CPF significantly damaged the intestinal mucosa, which was reversed after GSLS treatment.

**Figure 3:**
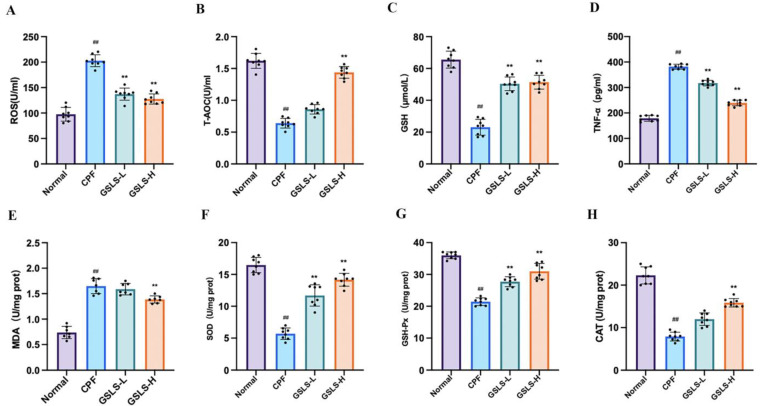
GSLSs alleviate CPF-induced intestinal toxicity and oxidative stress in mice. (**A**) MDA level in intestinal tissue. (**B**) SOD level in intestinal tissue. (**C**) GSH-Px level in intestinal tissue. (**D**) CAT level in intestinal tissue. (**E**) Serum ROS levels. (**F**) Serum T-AOC levels. (**G**) Serum GSH levels. (**H**) Serum TNF-α levels. All values are expressed as mean ± SD (*n* = 8); ^##^ *p* < 0.01 compared with the control group; ** *p* < 0.01 compared with the CPF group.

Compared to the control group, there was a significant decrease in the levels of GSH-Px, SOD and CAT in intestinal tissues after exposure to CPF, and the content of MDA was significantly increased. This suggests that the redox balance is disturbed due to CPF exposure. The findings indicate that CPF leads to intestinal oxidative stress in mice (Figure 3A–D). However, after 21 days of treatment with GSLSs (100 and 200 mg/kg), ROS, T−AOC, GSH, SOD and CAT levels in tissues were reversed. Interestingly, we found the same trend in the results of serum measurements of ROS, T-AOC and GSH (Figure 3E–G). These data confirm that GSLSs can ameliorate CPF-induced oxidative stress.

### 2.3. Effects of GSLSs on Serum Inflammatory Factors

In comparison to the control group, the level of the proinflammatory factor TNF-α was considerably elevated in the CPF group (all *p* < 0.05). Compared with the CPF group, the levels of the proinflammatory factor TNF-α in the GSLS administration groups (GSLS-L and GSLS-H) were significantly decreased (all *p* < 0.05). Among them, compared with the GSLS-H group, the level of the serum proinflammatory factor TNF−α in the GSLS-L group was significantly recovered (*p* < 0.05), as shown in Figure 3H.

### 2.4. Immunohistochemical Staining Analysis

**Figure 4:**
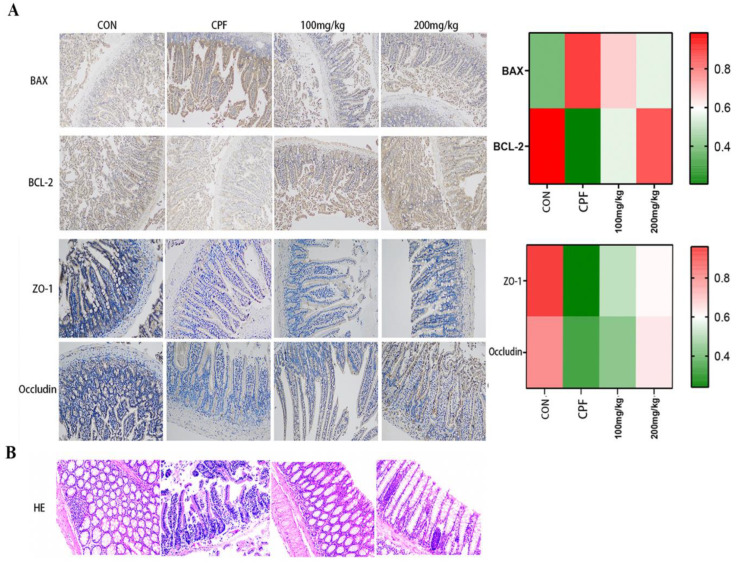
(**A**) The expression levels of Bcl-2, BAX, ZO-1 and Occludin in mouse intestinal tissues were detected by immunohistochemistry (200×). (**B**) H & E-stained images (200×) show the cross-sectional area of intestinal tissues in the control group, the CPF group, the GSLS-L group (100 mg/kg) and the GSLS-H group (200 mg/kg).

In this study, after CPF exposure, the degree of apoptosis in mouse intestinal tissues was assessed using immunohistochemical staining for Bcl-2 protein and proapoptotic protein Bax. In the CPF-exposed group, the protein level of small-intestinal Bax increased significantly, while the levels of Bcl-2, ZO-1, and Occludin decreased significantly. But in the GSLS treatment groups, Bax expression decreased while Bcl-2, ZO-1 and Occludin expression recovered (Figure 4A). In conclusion, GSLSs improved CPF-induced intestinal apoptosis by regulating the expression of Bcl-2/Bax proteins, and it simultaneously enhanced the integrity of the intestinal barrier and provided a protective effect.

### 2.5. Intestinal Histopathological Analysis

HE staining revealed that CPF exposure weakened the tight connections of the intestinal mechanical barrier, leading to reduced density and increased wrinkling of the intestinal barrier. Nonetheless, GSLSs at 100 and 200 mg/kg partially alleviated the intestinal damage caused by CPF exposure (Figure 4B).

### 2.6. GSLSs Reduced Phosphorylation, Inflammation and Apoptosis via IKKα/β and pIκBα in Intestinal Tissues

**Figure 5:**
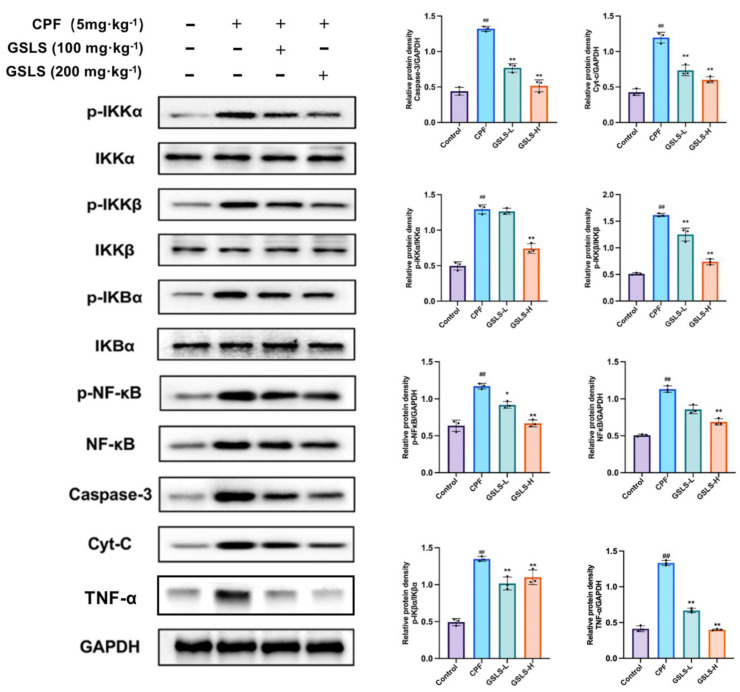
GSLSs improve CPF-induced enterotoxicity by reducing the phosphorylation of NF−κB. Western blotting was used to detect the protein levels of NF-κB, p-NF-κB, IκBα, p-IκBα, IKKα/β, p-IKKα/β, Caspase-3, Cyt-C and TNF-α in intestinal tissues and to quantify the expression of related proteins. ^##^ *p* < 0.01 compared with the control group; * *p* < 0.05 and ** *p* < 0.01 compared with the CPF group.

To further elucidate the anti-apoptotic activity of GSLSs in the mouse gut, Western blotting was conducted using anti-p-IκBα antibodies to visualize IκB phosphorylation under different treatments. The results showed that CPF-induced enterotoxicity was associated with the abnormal activation of IKKα/β, pIκBα and NF-κB protein expression. As shown in Figure 5, protein expression levels of p-IKKα/β and p-IκBα were significantly increased after CPF exposure compared to the control group. However, the aforementioned alterations were attenuated through the continuous administration of GSLSs for 3 weeks. Significantly, the GSLS-H group manifested a more potent therapeutic impact than the GSLS-L group (*p* < 0.05, *p* < 0.01).

In addition, compared with the control group, the expression levels of Cyt-c, Caspase-3 and TNF-α in the CPF group were significantly increased, indicating CPF-induced apoptosis. However, the expression levels of these indicators in the GSLS-H group showed a downward trend. The results showed that GSLSs could effectively slow down apoptosis in pesticide-poisoned mice. Taken together, these data suggest that GSLSs can ameliorate enterotoxicity by effectively inhibiting CPF-induced increases in IKKα/β and pIκBα phosphorylation in the mouse gut.

### 2.7. The Effect of GSLSs on CPF-Induced RAW264.7 Cell Model Using CCK-8

**Figure 6:**
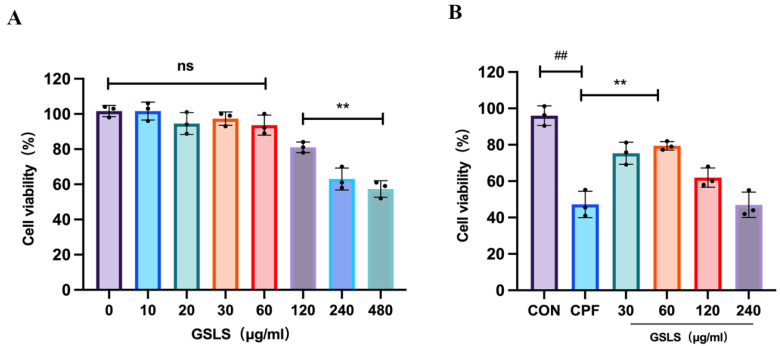
CCK-8 assay was used to detect cell viability (*n* = 3). (**A**) The effects of different concentrations of GSLSs on RAW264.7 cells were determined. (**B**) CCK-8 assay was used to determine the cell viability of RAW264.7 cells after CPF induction by GSLS. All values are expressed as mean ± SD. ^##^ *p* < 0.01 vs. the control group; ** *p* < 0.01 compared with the CPF group. ns, not significant.

Different concentrations of GSLSs were co-incubated with CPF-induced RAW264.7 cell models. As shown in Figure 6, cell viability was significantly reduced in the CPF group compared with the control group. When the GSLS concentration was 120–480 μg/mL, cell viability declined, with a significantly decreased density. When the GSLS concentration was 30–60 μg/mL, cell viability was significantly restored compared to the CPF group. Therefore, CPF concentrations of 30 μg/mL and 60 μg/mL were selected as dosages for subsequent experiments.

### 2.8. Analysis of Hoechst Staining Results

**Figure 7:**
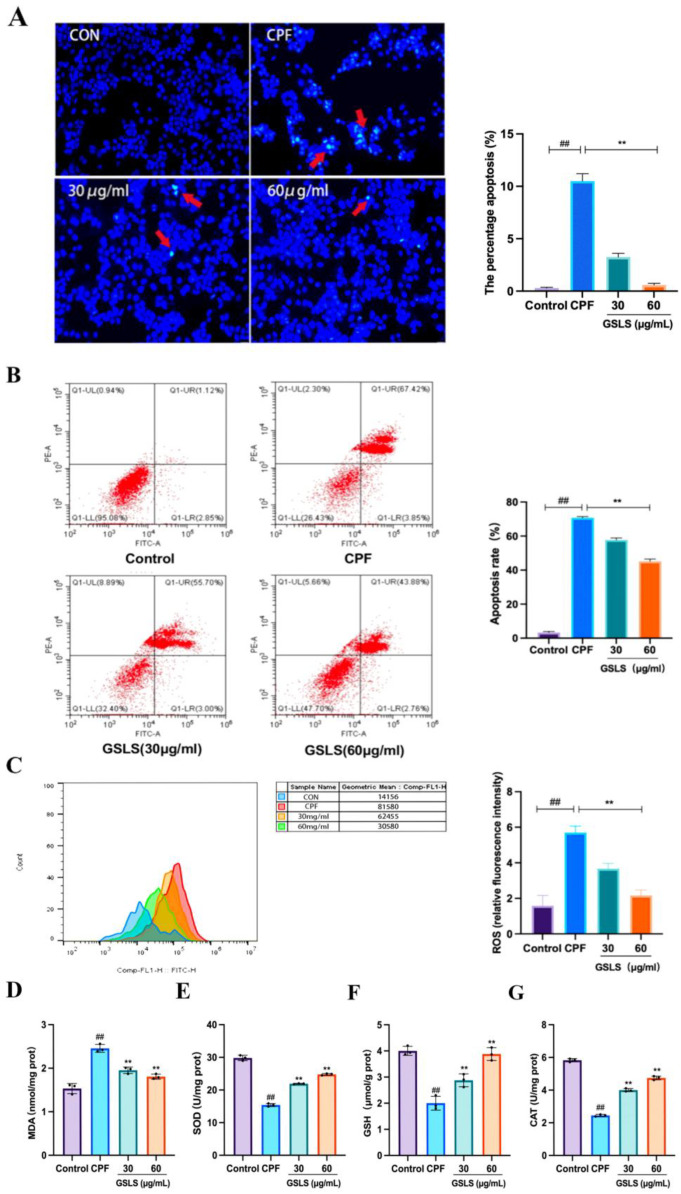
GSLSs improve CPF-induced enterotoxicity by inhibiting apoptosis, reducing ROS accumulation and ameliorating oxidative stress in RAW264.7 cells. (**A**) Hoechst 33258 staining (200×) and quantification of the percentage of apoptosis (%). (**B**) Flow cytometry was used to detect apoptosis in RAW-264.7 cells. (**C**) Intracellular ROS content was determined by flow cytometry. The levels of MDA (**D**), SOD (**E**), GSH (**F**) and CAT (**G**) (*n* = 3) were measured. All values are expressed as mean ± SD. ^##^ *p* < 0.01 vs. the control group; ** *p* < 0.01 compared with the CPF group.

Hoechst 33258 is a blue fluorescent stain that can traverse unperturbed cellular membranes. During the occurrence of apoptotic processes, the chromosomal DNA structure in apoptotic cells is changed, and the function of the P-glycoprotein pump on the membrane is impaired. Consequently, the Hoechst staining solution cannot be released from the affected cells, while the permeability of the cell membrane to the stain increases and the fluorescence intensity is strong. As shown in Figure 7A, a large number of bright blue spots were visible in the CPF group compared with the control group, exhibiting distinctive apoptotic characteristics. Conversely, bright blue spots were significantly reduced in the GSLS group compared to the CPF group. These findings suggest that GSLSs can improve CPF-induced apoptosis in RAW264.7 cells.

### 2.9. Inhibitory Effect of GSLSs on CPF-Induced Apoptosis in RAW264.7 Cells

Cell apoptosis was detected using flow cytometry. Figure 7B shows that in comparison to the control group, the percentage of apoptotic cells in the CPF group increased to 71.27%, and the apoptosis rate was reduced to 58.7% and 46.64% by GSLSs at concentrations of 30 μg/mL and 60 μg/mL, respectively. These findings indicate that GSLSs can effectively inhibit CPF-induced apoptosis in RAW264.7 cells, thus further affirming the in vivo results.

### 2.10. Effects of GSLSs on CPF-Induced Oxidative Stress in RAW264.7 Cells

ROS is a naturally occurring metabolite resulting from redox reactions. Nevertheless, an excessive level of ROS is strongly connected to the escalation and progression of oxidative stress damage. The intracellular quantity of ROS was gauged through DCFH-DA probes, which oxidized non-fluorescent DCFH and transformed it into fluorescent DCF. As shown in Figure 7C, cells in the CPF group produced a stronger DCF signal compared with the control group. After GSLS treatment, the fluorescence intensity decreased in a dose-dependent manner. In addition, the contents of MDA, SOD, GSH and CAT in RAW264.7 cells were determined to evaluate the effect of GSLSs on CPF-induced oxidative stress. The results in Figure 7D–G show that CPF significantly increased MDA and decreased SOD and CAT levels, along with GSH depletion, compared with the control group (*p* < 0.01, *p* < 0.05). However, a significant reduction in MDA and the recovery of GSH, SOD and CAT levels were observed in the GSLS treatment group (*p* < 0.01, *p* < 0.05). The above data indicate that GSLSs have the potential to significantly reduce ROS accumulation and alleviate CPF-induced oxidative stress damage in RAW264.7 cells.

### 2.11. GSLSs Ameliorated CPF-Induced Inflammatory Factors in RAW264.7 Cells

**Figure 8:**
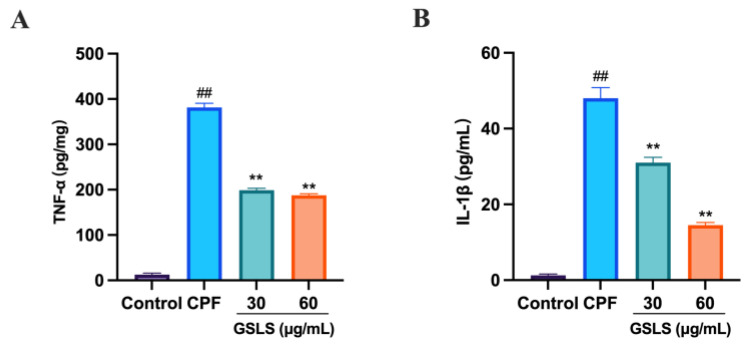
Effect of GSLSs on inflammatory factors in CPF-induced RAW264.7 cells. The levels of TNF-α (**A**) and IL-1β (**B**). All values are expressed as mean ± SD. (*n* = 3). ^##^ *p* < 0.01 vs. the control group; ** *p* < 0.01 compared with the CPF group.

To determine the correlation between CPF-induced intestinal damage and inflammation, the levels of the inflammatory factors TNF−a and IL−1β levels were measured. As shown in Figure 8A,B, GSLS administration significantly reduced the CPF-induced increases in TNF-α and IL-1β levels compared with the CPF group (*p* < 0.01, *p* < 0.05). These findings suggest that GSLSs have an anti-inflammatory effect in ameliorating CPF-induced intestinal damage.

## 3. Discussion

After contact with OPs, the absorption of toxins can lead to organ function damage in organisms, and in severe cases, systemic organ failure or even death may also occur [42]. Currently, there are many extensive studies on first aid for organophosphorus poisoning. For example, the antibiotics atropine and phosphoramidonium chloride can reactivate the inhibited cholinesterase [32,43]. Blood purification techniques are effective in removing gastrointestinal toxins, as are antioxidant reductants [44]. However, the clinical dosing, protection and prevention of intestinal toxicity and damage by OP-type pesticides still need further research.

CPF (chemical name O, O-diethyl hydrogen phosphorothioate) is a broad-spectrum organophosphate insecticide developed by Dow Chemical Company. At present, OP is one of the most used pesticides in Chinese agricultural production and is also the main pesticide causing acute poisoning and organ damage [45,46]. After CPF enters the organism, it first destroys the intestinal mucosa, causing intestinal mucosal wall atrophy, digestive stress ulcers and increased intestinal barrier permeability [47], and at the same time, it causes oxidative stress in the intestinal tract and induces enterotoxicity. In this study, CPF-induced RAW264.7 macrophages were used to mimic in vitro intestinal macrophage inflammation, oxidative stress and apoptosis, and C57BL/6J mice were used to establish an in vitro animal model of intestinal injury.

The worsening of enterotoxicity after pesticide exposure is usually characterized by oxidative stress and persistent inflammation [48,49]. Therefore, antioxidant and anti-inflammatory pathways are ways to ameliorate enterotoxicity [50]. The intestinal tract is one of the organs capable of producing large amounts of free radicals. Clinical studies have shown that increased lipid peroxidation products in patients with organophosphorus pesticide poisoning can further aggravate tissue damage [51]. In the present study, by decreasing the levels of oxidative-stress-related indicators such as SOD, CAT, ROS, T-AOC, GSH and GSH-Px and reducing the content of lipid peroxide MDA, we confirmed that the activity of GSLSs against CPF-induced intestinal damage was achieved by inhibiting oxidative stress, which, to a certain extent, proves that GSLSs have the potential to be natural antioxidants for intestinal cells.

Nuclear factor-κ gene binding (NF-κB) is an important transcription factor for the activation of many inflammatory mediators, most of which exist in the form of heterodimers [52], which bind to inhibitory IκB proteins (IκBα and IκBβ) in the cytoplasm [53]. However, under inflammatory conditions, IκB is phosphorylated by IκB kinase (IKK) and subsequently degraded by the proteasome [54]. Subsequently, NF-κB is released and translocated to the nucleus, where it triggers the transcription of multiple genes via the cis-acting element κB [55].

The results of our previous studies suggested that CPF-induced ROS accumulation mediates oxidative stress damage and interferes with mitochondrial membrane potential homeostasis and cell function [37]. Wang et al. [56] showed that ROS accumulation in RAW264.7 cells is an upstream signal to mobilize the NF-κB pathway and causes the overproduction of inflammatory factors (TNF-α, IL-1β). NF-κB can promote the expression of various proinflammatory cytokines, such as TNF-α [57,58]. The results of this study showed that compared with the control group, the levels of TNF-α and IL-1β in the intestinal tissues of the CPF group were significantly increased. The anti-inflammatory effect of GSLSs was also associated with the inhibition of the production of such proinflammatory factors in intestinal tissues in our study. NF-κB activates TNF-α during inflammation and, subsequently, the inflammatory cascade of CPF-induced organ damage in mice.

The activation of NF-κB in the inflammatory signaling pathway is associated with the overproduction of ROS [59]. NF-κB plays a key role in regulating cell proliferation, the inflammatory response, survival and apoptosis. The main component of IκB is IκBα, the first protein to be described in the IκB family and the most extensively studied IκB protein to date. As a dominant regulatory kinase in the classical NF-κB signaling pathway, IκBα also plays a prominent role in apoptosis. The activation of NF-κB may be a key event in proinflammatory signal transduction. In the present study, we speculated that the palliative effect of GSLSs may be related to the inflammatory response associated with the activation of NF-κB. To elucidate these mechanisms, we detected the expression of p-IKKα/β and p-IκBα. Importantly, the results showed that CPF induced and triggered a large amount of NF-κB and IκBα nuclear translocation, while IκBα did not degrade. GSLSs attenuated the abnormal activation of the NF-κB pathway induced by CPF. For BAX/Bcl-2 proteins associated with apoptosis, GSLSs attenuated the expression of Bax and reversed the expression of Bcl-2 protein. GSLSs have good protective and palliative effects on CPF-induced oxidative damage and can reduce the enterotoxicity caused by CPF exposure by reducing inflammation and improving apoptosis.

It is well known that a significant increase in ROS in intestinal macrophages under pathological conditions is a direct cause of intestinal barrier dysfunction and enterotoxicity [60]. The results of the data suggest that RAW264.7 cells showed excessive accumulation of ROS after CPF treatment, which ultimately disrupted the normal physiological function of the intestinal barrier. Interestingly, all of these conditions were reversed after GSLS treatment.

Diamine oxidase (DAO) is an enzyme in intestinal epithelial cells, and changes in its activity are strongly associated with damage to and repair of intestinal mucosal epithelial cells [61]. Therefore, the level of DAO in intestinal tissues was used as an important indicator to determine intestinal injury. Our results showed that the DAO content in the intestinal tissues of mice in the CPF group was significantly increased. In the GSLS group (GSLS-L and GSLS-H), the DAO content was significantly reduced. Meanwhile, histopathological findings showed that mice in the CPF group had intestinal epithelial damage and cryptic aberrations. However, after GSLS treatment, the intestinal damage was significantly improved. The levels of DAO in intestinal tissues were dose-dependently reduced after the administration of GSLS.

Intestinal tight-junction proteins are associated with the integrity of the intestinal epithelial barrier and the homeostasis of intestinal cells. Once the intestinal tight-junctions are disrupted by exogenous toxic substances, the permeability and integrity of the intestinal barrier can be compromised and even potentially harmful to the organism [25,62]. In this study, when we induced apoptosis in RAW264.7 cells using CPF, we observed the weak expression of Bcl-2 protein and the attenuated expression of the tight-junction proteins Occludin and ZO-1. This suggests that GSLSs can ameliorate CPF-induced enterotoxicity by inhibiting ROS-mediated apoptosis, upregulating the levels of tight-junction proteins and protecting the intestinal epithelial barrier.

In recent years, many researchers have focused on the in-depth study and exploration of the pathogenesis of major diseases at the level of apoptosis regulation [63,64,65]. Thus, the inhibition of apoptotic hyperactivity has the potential to ameliorate the pathological changes in a variety of apoptosis-related diseases. Notably, apoptosis also seems to play a crucial role in intestinal oxidative stress injury and enterotoxicity [66].

Intracellular anti-apoptosis depends, to some extent, on the levels of anti-apoptotic Bcl-2 and proapoptotic Bax proteins. Both Bax and Bcl-2 are family members of B-cell lymphoma 2 (Bcl-2) and play major roles in spreading or inhibiting intrinsic apoptotic pathways [67]. A large number of free anti-apoptotic Bcl-2 family members are present in healthy cells to bind and alienate proapoptotic Bax [68]. However, Bax is released, which is a marker of apoptosis. Therefore, the ratio of Bax/Bcl-2 is closely related to the occurrence of apoptosis. In the case of intestinal oxidative stress injury, we believe that the effective regulation of the Bax/Bcl-2 ratio is also an effective therapeutic route [69]. The release of Cyt-c is considered to be a key event in apoptosis [70]. ROS deposition caused by oxidative stress in an organism can directly lead to the release of precursors of Cyt-c and caspases from mitochondria to the cytoplasm. Meanwhile, Cyt-c released by mitochondria can activate caspase-9, activate caspase-3, and ultimately lead to intestinal cell death. Bcl-2 family proteins can prevent the release of Cyt-c and inhibit apoptosis, while Bax can induce the release of Cyt-c and promote apoptosis [47,71]. Studies have shown that the activation of Bax due to oxidative stress leads to the release of Cyt-c from mitochondria to the cytoplasm and promotes the activation of Pro-Caspase-3 to Caspase-3, thus triggering the process of apoptosis [72].

## 4. Materials and Methods

### 4.1. Materials

Hematoxylin and eosin (H&E) and ELISA kits for the determination of CAT, MDA, SOD, GSH, GSH-Px, T-AOC, TNF-α, IL-1β and DAO were purchased from Beijing Sorobio Technology Co., Ltd. (Beijing, China). 2,7-Dichlorofluorescein diacetate (DCFH-DA) and BCA kits were from Beyotime Biotechnology; primary antibodies targeting ZO-1, Occludin, IκB kinase α/β (IKKα/β), κBα inhibitor (IκBα), NF-κB, p-IKKα, p-IKKβ, phosphorylated IκBα (p-IκBα), phosphorylated NF-κB (p-NF-κB), cytochrome C (Cyt-C), caspase-3, TNF-α and GAPDH, rabbit anti-Bax antibody and rabbit anti-Bcl-2 L1 antibody were obtained from Guangzhou Sulai Biotechnology Co., Ltd. (Guangzhou, China).

CPF was purchased from Kaifeng Inspur Chemical Co., Ltd. (Henan, China). To avoid possible inhibition of CPF by serum proteins, cells were treated with CPF dissolved in a medium containing 1% fetal bovine serum. Dimethyl sulfur oxide (DMSO) was used as a solvent to dilute insecticides (Beijing Sorobio Technology Co., Ltd., Beijing, China). The concentration of DMSO remained below 0.05% during the experiment. The saline solution was purchased from Heilongjiang Kelun Pharmaceutical Co., Ltd. (Heilongjiang, China), and the CCK-8 kit, ROS kit and PI/Accessory v dye were purchased from the company. The Hoechst 33258 staining kit and Annexin V fitc PI were from Shanghai Bioscience & Technology Co., Ltd. (Shanghai, China). All other chemicals were analytical-grade and obtained from commercial sources. GSLSs were provided by Jilin University in Changchun, Jilin, China.

### 4.2. Experimental Design

#### 4.2.1. HPLC Analysis

GSLS samples were analyzed on a Waters e2695 HPLC system (Waters Corporation, Milford, CT, USA) equipped with a UV detector. A Waters C18 column (4.6 cm × 25 cm, 5 μm) was isolated and analyzed at 30 °C, and the detection wavelength was set at 210 nm. The mobile phase consisted of a mixture of acetonitrile (A) and water (B) and was set in 4Q [66]. Gradient elution procedure: 0–30 min, 18.0–25.0% A; 30–60 min, 25.0–27.0% A; 60–70 min, 27.0–27.0% A; 70–71 min, 27.0–18% A. The flow rate was 1.0 mL/min. The injection volume was 20 μL.

#### 4.2.2. In Vivo Animal Experiments and Design

Experimental animals were provided by Changchun YISI Experimental Animals Co., Ltd. (Changchun, China) with a Certificate of Quality (SCXK (JI)-2018-0023). Eight-week-old male C57BL/6J mice weighing 20–22 g were used. The mice were housed for 7 days in a 12 h light–dark cycle to acclimate to the environment, during which the mice were kept at constant temperature (22 ± 2 °C) and had free access to standard food and drinking water. All animals were cared for in accordance with Animal Care and Use guidelines. All experimental procedures were approved by the Experimental Animal Ethics Committee of Jilin Agricultural University and the Animal Protection Work Committee of Jilin Agricultural University (2016-01) (License No. ECLA-JLAU 2016-016) (No. 2020 11 18 001). The experiment was designed to establish an animal model of intestinal injury exposed to CPF. Both the grouping and the subsequent treatment followed the randomization principle. The mice were randomly divided into 4 groups (*n* = 8): the control, CPF (CPF, 5 mg/kg), GSLS-L (CPF + GSLS-100 mg/kg) and GSLS-H (CPF + GSLS-200 mg/kg). Except for the control group, the mice in both the GSLS-L and GSLS-H groups were preprotected by GSLS gavage for 1 week, and then all mice were gavaged with CPF continuously for 1 week to establish the intestinal damage model of CPF (the control group was gavaged with the same amount of normal saline). Both groups were given GSLSs by gavage for 21 days. On the 35th day, the experiment was terminated, and all mice were anesthetized with dimethyl ether and sacrificed by neck dissection. The fresh small-intestine tissue, blood and organ samples were immediately collected for analysis, as shown in Figure 2A. Serum and tissue samples were stored in an ultra-low-temperature refrigerator at −80 °C for subsequent testing.

#### 4.2.3. Analysis of Serum Oxidative Stress Index

A Bio-Rad enzyme-linked immunosorbent assay (ELISA) was used to determine the activities of ROS, T-AOC and GSH in the serum of mice. Serum was separated from collected blood by 1000× *g* centrifugation twice, each time for 10 min. According to the protocol provided by the manufacturer of the ELISA reader at 450 nm, the sample was added to a 96-well plate coated with mouse ROS-, T-AOC- and GSH-specific antibodies.

#### 4.2.4. DAO and Biochemical Analysis of Intestinal Tissue

Mouse intestinal tissues were used to evaluate DAO and antioxidant activities. Lipid peroxides were measured by MDA kits, and SOD and CAT activities and GSH-Px content were measured according to the kit manufacturer’s instructions. According to the manufacturer of the ELISA reader provided in the protocol, samples were added to the microplate coated with the specific antibody, and then OD was read at 450 nm.

#### 4.2.5. The Influence of Proinflammatory Cytokines Was Determined by ELISA

The serum of mice was collected, and according to the instructions of the kit, the serum proinflammatory factor TNF-α was detected. The optical density value was determined using the enzyme marker, and then the index level was calculated using the standard curve.

#### 4.2.6. Immunohistochemical

Small-intestine tissues were fixed in 10% formaldehyde and embedded in paraffin for 24 h prior to subsequent processing. The paraffin-embedded tissues were prepared at a thickness of 5 μM, and sections of small-intestine tissues were incubated in PBS for 5 min and washed in the same buffer 3 times. The slices were then incubated in 1% hydrogen peroxide (H_2_O_2_) for 30 min to block endogenous peroxidase activity. After washing in PBS, sections were incubated in sealed serum (10% normal goat serum and 0.1% Triton X-100 in PBS) for 2 h. They were then incubated overnight with murine monoclonal antibodies against Bcl-2, Bax, ZO-1 and Occludin. Next, the sections were incubated with the antibiotin protein-biotin peroxidase complex at room temperature for 1 h. They were then incubated in a staining solution consisting of 0.02% diaminobenzidine tetramine hydrochloride for immunohistochemistry using the antibiotin protein-biotin technique. Hematoxylin staining was used as a reverse staining procedure. The mean optical density (IOD) of protein expression was measured using an Image J image analysis system (Image-Pro plus 6.0, Media Cybernetics, Inc., Rockville, MD, USA), and differences in positive protein expression levels between groups were calculated.

#### 4.2.7. HISTOPATHOLOGICAL Examination

The pathological changes in the intestinal tracts of mice were observed with a microscope. Histopathological sections were randomly selected and analyzed under a microscope (Leica, DM2500, Wetzlar, Germany) at 200× magnification, and pathological changes were observed, including intestinal villi shedding, cell necrosis, and density changes.

Briefly, mouse intestinal tissue was collected and then washed with a phosphate-buffered saline solution. Specifically, the intestinal tissues were fixed in 4% paraformaldehyde for 24 h, embedded in paraffin, and cut into sections with a thickness of 5 μM. Histopathological changes were fixed with a neutral gel and subsequently stained with HE according to the manufacturer’s instructions. Representative images were taken using a light microscope (Olympus BX-60, Tokyo, Japan).

#### 4.2.8. Protein Imprinting Detection

The intestinal tissues of mice were fully lysed by Radio Immunoprecipitation Assay (RIPA) buffer, and 0.1 g of intestinal tissue was homogenized in 1 mL of cold RIPA buffer. The manufacturer’s instructions were followed to determine protein concentration using the BCA kit. The same amount of protein was isolated by SDS-PAGE and transferred to a polyvinylidene fluoride membrane. The membrane was blocked with TBS-T (pH 8.0) with 0.5% BSA for 1.5 h and then incubated at 4 °C overnight with the appropriate dilution of primary antibodies against p-IκBα, p-IKKα/β, IκBα, IKKα/β, NF-κB, p-NF-κB, Cyt-c, Caspase-3 and TNF-α. The expression of GAPDH was used to show the same protein load. After the PVDF membranes were rinsed with TBS-T 3 times (10 min each time), they were incubated with secondary antibodies coupled with horseradish peroxidase (HRP) at room temperature for 1 h. Finally, the blot was incubated with ECL (Proteintech, Chicago, IL, USA). The software Image J (Image-Pro plus 6.0, Media Cybernetics, Inc., Rockville, MD, USA) was used to quantify the gray levels of the bands.

### 4.3. In Vitro Cell Culture and Treatment

RAW264.7 cells were cultured in DMEM medium supplemented with 1% penicillin/streptomycin (10.000 U/mL; 10 mg/mL) and amphotericin B (0.25 mg/mL) and heat-inactivated fetal bovine serum (FBS) at 10% final concentration. Until the desired number of cells and confluence were reached for subsequent experiments, RAW264.7 cells in the exponential growth stage were used in this experiment.

#### 4.3.1. CCK-8 Detection of Cell Viability

RAW264.7 cells were inoculated on 96-well culture plates at a density of 1 × 10^5^ cells/well and grew at 37 °C for 12 h. The experiment was divided into the control, CPF (1 μg/mL), GSLS-L and GSLS-H groups. The cells were inoculated in 96-well culture plates. Except for the control group, all other groups (10, 20, 30, 60, 120, 240 and 480 μg/mL GSLSs) were treated with 1 μg/mL CPF for 12 h. Cell viability was measured using CCK-8. To each well, 10 μL of CCK-8 solution was added, followed by incubation at 37 °C for 2 h, and absorption was measured at 450 nm with Epoch2 (Burton Instruments, Inc., Gainesville, FL, USA).

Cell viability (%) was calculated as follows:

Cell viability (100%) = (A_drug_ − A_blank_)/(A_control_ − A_blank_) × 100%

#### 4.3.2. Assessment of Biochemical Parameters

The oxidation indexes GSH, MDA, CAT and SOD and the inflammatory factors TNF-α and IL-1β were determined according to the manufacturer’s instructions. RAW264.7 cells were inoculated into a 6-well culture plate and incubated until the cells reached 70% confluence. After modeling with CPF (1 μg/mL) and administration of GSLSs, the medium was discarded, and the cells were washed twice with PBS. The scraped cells were collected, re-suspended in 1 mL of PBS, and then homogenized to destroy the cell membrane. At 3000× *g* and 4 °C for 10 min, the supernatant was collected, and immediately, biochemical determination was performed. Absorbance was measured with a SPECTROstar Nano (Ottenberg, Germany).

#### 4.3.3. Hoechst 33258 Staining

The apoptotic morphology of RAW264.7 cells was detected using the Hoechst 33258 staining kit according to the kit company’s instructions. Cells were divided into the following groups: the control group, the CPF (1 μg/mL) group and two GSLS groups with a dose of CPF at 1 μg/mL and with doses of GSLSs at 30 and 60 μg/mL, respectively, co-incubated as a treatment group. RAW264.7 cells were washed three times with PBS, incubated with Hoechst 33258 staining solution for 20 min in an incubator at 37 °C, and washed three more times with PBS. The excitation wavelength was 350 nm, and the emission wavelength was 460 nm for fluorescence microscopy.

#### 4.3.4. Apoptosis Was Detected by Flow Cytometry

According to the results of cell culture, apoptosis was detected by flow cytometry. The treated cells were collected and centrifuged at 400× *g* for 5 min and washed twice with PBS. The cell precipitate was collected, and buffer was added to prepare a 1 × 10^6^ cell suspension. According to the manufacturer’s instructions, Annexin V and PI dye were added to the cell suspension separately and incubated at room temperature in the dark for 10 and 5 min, respectively. Then, apoptosis was analyzed with the CytoFLEXLX flow cytometer (Beckman Coulter, Inc., Brea, CA, USA).

#### 4.3.5. ROS Detection

The intracellular ROS level was measured using a fluorescent probe, DCFH-DA. RAW264.7 cells were inoculated in 6-well plates at a density of 1 × 10^5^. After 12 h of culture in complete medium, the cells were cleaned twice with PBS and incubated with 10 μM DCFH-DA at 37 °C for 20 min. The cells were then washed three times with PBS. The fluorescence intensity of intracellular ROS was measured by flow cytometry.

#### 4.3.6. Statistics and Analysis

All experimental data in this experiment were statistically analyzed using GraphPad Prism 9.0 software (USA). A one-way ANOVA model was used to compare two or more groups of experimental data, and then a Bonferroni post-test was carried out. All experimental data are expressed as mean ± standard deviation (standard definition). *p* < 0.05 was considered statistically significant.

## 5. Conclusions

Our findings demonstrate that GSLSs reversed CPF-induced oxidative stress and inflammation and inhibited the abnormal activation of proinflammatory protein pathways, thereby attenuating the intestinal damage caused by CPF exposure. The results showed that GSLSs reduced intestinal barrier injury, increased the expression of the tight-junction proteins ZO-1 and Occludin, restored the expression of the anti-apoptotic protein Bcl-2 and decreased the expression of the proapoptotic protein Bax, thus protecting the intestinal tract from CPF. In addition, it was also found in in vitro experiments that GSLSs attenuated CPF-induced ROS accumulation and apoptotic injury in RAW264.7 cells. The above findings provide a theoretical basis for the prevention and control of CPF-exposure-induced enterotoxicity and provide scientific support for the future clinical application of GSLSs. Since only in vivo and in vitro experiments on CPF were conducted in this study, and there are many types of OP-type organophosphorus pesticides and their toxicity pathways may be different, future research directions or application prospects need to be further explored. We hope to provide more useful evidence for clinical treatment and drug development.

## Data Availability

The data that support the findings of this study are available from the corresponding author upon reasonable request.

## References

[1] Paidi M.K., Satapute P., Haider M.S., Udikeri S.S., Ramachandra Y.L., Vo D.-V.N., Govarthanan M., Jogaiah S. (2021). Mitigation of organophosphorus insecticides from environment: Residual detoxification by bioweapon catalytic scavengers. Environ. Res..

[2] Silva J., Marques-Da-Silva D., Lagoa R. (2021). Reassessment of the experimental skin permeability coefficients of polycyclic aromatic hydrocarbons and organophosphorus pesticides. Environ. Toxicol. Pharmacol..

[3] Sarlak Z., Khosravi-Darani K., Rouhi M., Garavand F., Mohammadi R., Sobhiyeh M.R. (2021). Bioremediation of organophosphorus pesticides in contaminated foodstuffs using probiotics. Food Control.

[4] Millis R.M., Archer P.W., Whittaker J.A., Trouth C.O. (1988). The role of hypoxia in organophosphorus nerve agent intoxication. Neurotoxicology.

[5] Sidhu G.K., Singh S., Kumar V., Dhanjal D.S., Datta S., Singh J. (2019). Toxicity, monitoring and biodegradation of organophosphate pesticides: A review. Crit. Rev. Environ. Sci. Technol..

[6] Rosenbaum C., Bird S.B. (2010). Non-muscarinic Therapeutic Targets for Acute Organophosphorus Poisoning. J. Med. Toxicol..

[7] Guignet M., Dhakal K., Flannery B.M., Hobson B.A., Zolkowska D., Dhir A., Bruun D.A., Li S., Wahab A., Harvey D.J. (2020). Persistent behavior deficits, neuroinflammation, and oxidative stress in a rat model of acute organophosphate intoxication. Neurobiol. Dis..

[8] Raszewski G., Filip R. (2004). Use of oximes in the therapy of acute intoxication by organophosphorus compounds. Prz. Lek..

[9] Abdollahi M., Karami-Mohajeri S. (2012). A comprehensive review on experimental and clinical findings in intermediate syndrome caused by organophosphate poisoning. Toxicol. Appl. Pharmacol..

[10] Wang G., Li J., Xue N., Al-Huqail A.A., Majdi H.S., Darvishmoghaddam E., Assilzadeh H., Khadimallah M.A., Ali H.E. (2022). Risk assessment of organophosphorus pesticide residues in drinking water resources: Statistical and Monte-Carlo approach. Chemosphere.

[11] Jin S., Zhu T., Deng S., Li D., Li J., Liu X., Liu Y. (2022). Dioscin ameliorates cisplatin-induced intestinal toxicity by mitigating oxidative stress and inflammation. Int. Immunopharmacol..

[12] Li D., Huang Q., Lu M., Zhang L., Yang Z., Zong M., Tao L. (2015). The organophosphate insecticide chlorpyrifos confers its genotoxic effects by inducing DNA damage and cell apoptosis. Chemosphere.

[13] Huang H.-M., Pai M.-H., Liu J.-J., Yeh S.-L., Hou Y.-C. (2019). Effects of dietary exposure to chlorpyrifos on immune cell populations and inflammatory responses in mice with dextran sulfate sodium-induced colitis. Food Chem. Toxicol..

[14] Sies H. (2015). Oxidative stress: A concept in redox biology and medicine. Redox Biol..

[15] Shang X., Xu W., Zhang Y., Sun Q., Li Z., Geng L., Teng X. (2023). Transcriptome analysis revealed the mechanism of *Luciobarbus capito* (*L. capito*) adapting high salinity: Antioxidant capacity, heat shock proteins, immunity. Mar. Pollut. Bull..

[16] Wang Z., Wei D., Xiao H. (2013). Methods of Cellular Senescence Induction using Oxidative Stress. Biological Aging.

[17] Zhao C., Teng X., Yue W., Suo A., Zhou W., Ding D. (2023). The effect of acute toxicity from tributyltin on *Liza haematocheila* liver: Energy metabolic disturbance, oxidative stress, and apoptosis. Aquat. Toxicol..

[18] Prajapati P., Gohel D., Shinde A., Roy M., Singh K., Singh R. (2020). TRIM32 regulates mitochondrial mediated ROS levels and sensitizes the oxidative stress induced cell death. Cell. Signal..

[19] Scherz-Shouval R., Elazar Z. (2011). Regulation of autophagy by ROS: Physiology and pathology. Trends Biochem. Sci..

[20] Zhou Q., Cui J., Liu Y., Gu L., Teng X., Tang Y. (2023). EGCG alleviated Mn exposure-caused carp kidney damage via trpm2-NLRP3-TNF-α-JNK pathway: Oxidative stress, inflammation, and tight junction dysfunction. Fish Shellfish Immunol..

[21] Jiao W., Han Q., Xu Y., Jiang H., Xing H., Teng X. (2019). Impaired immune function and structural integrity in the gills of common carp (*Cyprinus carpio* L.) caused by chlorpyrifos exposure: Through Oxidative Stress Apoptosis. Fish Shellfish Immunol..

[22] Li X., Bai Y., Bi Y., Wu Q., Xu S. (2023). Baicalin suppressed necroptosis and inflammation against chlorpyrifos toxicity; involving in ER stress and oxidative stress in carp gills. Fish Shellfish Immunol..

[23] Wang T., Ma M., Chen C., Yang X., Qian Y. (2021). Three widely used pesticides and their mixtures induced cytotoxicity and apoptosis through the ROS-related caspase pathway in HepG2 cells. Food Chem. Toxicol..

[24] Lee Y., Kamada N., Moon J.J. (2021). Oral nanomedicine for modulating immunity, intestinal barrier functions, and gut microbiome. Adv. Drug Deliv. Rev..

[25] Shi X., Xu W., Che X., Cui J., Shang X., Teng X., Jia Z. (2023). Effect of arsenic stress on the intestinal structural integrity and intestinal flora abundance of *Cyprinus carpio*. Front. Microbiol..

[26] Xing T., Benderman L.J., Sabu S., Parker J., Yang J., Lu Q., Ding L., Chen Y.-H. (2020). Tight Junction Protein Claudin-7 Is Essential for Intestinal Epithelial Stem Cell Self-Renewal and Differentiation. Cell. Mol. Gastroenterol. Hepatol..

[27] Miao Z., Miao Z., Teng X., Xu S. (2023). Melatonin alleviates lead-induced fatty liver in the common carps (*Cyprinus carpio*) via gut-liver axis. Environ. Pollut..

[28] Scaldaferri F., Pizzoferrato M., Gerardi V., Lopetuso L., Gasbarrini A. (2012). The gut barrier: New acquisitions and therapeutic approaches. J. Clin. Gastroenterol..

[29] Qin W., Xu B., Chen Y., Yang W., Xu Y., Huang J., Duo T., Mao Y., Zhou G., Yan X. (2022). Dietary ellagic acid supplementation attenuates intestinal damage and oxidative stress by regulating gut microbiota in weanling piglets. Anim. Nutr..

[30] Yan M., Zhu L., Wu S., Cao Y., Mou N., Chi Q., Wang G., Zhong Y., Wu W. (2022). ROS responsive polydopamine nanoparticles to relieve oxidative stress and inflammation for ameliorating acute inflammatory bowel. Biomater. Adv..

[31] Cui J., Hao Z., Zhou Q., Qiu M., Liu Y., Liu Y., Teng X., Kang L. (2023). Chlorpyrifos induced autophagy and mitophagy in common carp livers through AMPK pathway activated by energy metabolism disorder. Ecotoxicol. Environ. Saf..

[32] Thiermann H., Steinritz D., Worek F., Radtke M., Eyer P., Eyer F., Felgenhauer N., Zilker T. (2011). Atropine maintenance dosage in patients with severe organophosphate pesticide poisoning. Toxicol. Lett..

[33] Mitra J.K., Hansda U., Bandyopadhyay D., Sarkar S., Sahoo J. (2023). The role of a combination of N-acetylcysteine and magnesium sulfate as adjuvants to standard therapy in acute organophosphate poisoning: A randomized controlled trial. Heliyon.

[34] Thakur A., Patwa J., Pant S., Flora S.J.S., Sharma A. (2022). Synthesis and evaluation of small organic molecule as reactivator of organophosphorus inhibited acetylcholinesterase. Drug Chem. Toxicol..

[35] Wolthuis O.L., Philippens I.H.C.H.M., Vanwersch R.A.P. (1989). Side effects of therapeutic drugs against organophosphate poisoning. Neurotoxicol. Teratol..

[36] Potenza M.A., Montagnani M., Santacroce L., Charitos I.A., Bottalico L. (2022). Ancient herbal therapy: A brief history of *Panax ginseng*. J. Ginseng Res..

[37] Liu S., Pei H., Chen W., Zhu X., Wang Y., Li J., He Z., Du R. (2023). Evaluating the effect of ginsenoside Rg1 on CPF-induced brain injury in mice via PI3k/AKT pathway. J. Biochem. Mol. Toxicol..

[38] Aravinthan A., Kim J.H., Antonisamy P., Kang C.-W., Choi J., Kim N.S., Kim J.-H. (2015). Ginseng total saponin attenuates myocardial injury via anti-oxidative and anti-inflammatory properties. J. Ginseng Res..

[39] Ahn S., Siddiqi M.H., Noh H.-Y., Kim Y.-J., Kim Y.-J., Jin C.-G., Yang D.-C. (2015). Anti-inflammatory activity of ginsenosides in LPS-stimulated RAW 264.7 cells. Sci. Bull..

[40] de Oliveira Zanuso B., de Oliveira dos Santos A.R., Miola V.F.B., Campos L.M.G., Spilla C.S.G., Barbalho S.M. (2022). *Panax ginseng* and aging related disorders: A systematic review. Exp. Gerontol..

[41] Zhang Q.H., Wu C.F., Duan L., Yang J.Y. (2008). Protective effects of total saponins from stem and leaf of *Panax ginseng* against cyclophosphamide-induced genotoxicity and apoptosis in mouse bone marrow cells and peripheral lymphocyte cells. Food Chem. Toxicol..

[42] Zhang F., Tang S., Zhao L., Yang X., Yao Y., Hou Z., Xue P. (2020). Stem-leaves of Panax as a rich and sustainable source of less-polar ginsenosides: Comparison of ginsenosides from *Panax ginseng*, American ginseng and *Panax notoginseng* prepared by heating and acid treatment. J. Ginseng Res..

[43] Eddleston M., Buckley N.A., Eyer P., Dawson A.H. (2008). Management of acute organophosphorus pesticide poisoning. Lancet.

[44] Jiang S.-Z., Ma B.-E., Liu C., Wang R. (2019). Clinical efficacy of intravenous infusion of atropine with micropump in combination with hemoperfusion on organophosphorus poisoning. Saudi J. Biol. Sci..

[45] Yao M., Nie H., Yao W., Yang X., Zhang G. (2022). A sensitive and selective fluorescent probe for acetylcholinesterase: Synthesis, performance, mechanism and application. Arab. J. Chem..

[46] Rathod A.L., Garg R. (2017). Chlorpyrifos poisoning and its implications in human fatal cases: A forensic perspective with reference to Indian scenario. J. Forensic Leg. Med..

[47] Cui J., Liu Y., Hao Z., Liu Y., Qiu M., Kang L., Teng X., Tang Y. (2023). Cadmium induced time-dependent kidney injury in common carp via mitochondrial pathway: Impaired mitochondrial energy metabolism and mitochondrion-dependent apoptosis. Aquat. Toxicol..

[48] Vismaya, Rajini P. (2014). Oral exposure to the organophosphorus insecticide, Monocrotophos induces intestinal dysfunction in rats. Food Chem. Toxicol..

[49] Alruhaimi R.S. (2023). Betulinic acid protects against cardiotoxicity of the organophosphorus pesticide chlorpyrifos by suppressing oxidative stress, inflammation, and apoptosis in rats. Environ. Sci. Pollut. Res..

[50] Abolhassani M., Asadikaram G., Paydar P., Fallah H., Aghaee-Afshar M., Moazed V., Akbari H., Moghaddam S.D., Moradi A. (2019). Organochlorine and organophosphorous pesticides may induce colorectal cancer; A case-control study. Ecotoxicol. Environ. Saf..

[51] Zhao G.-P., Wang X.-Y., Li J.-W., Wang R., Ren F.-Z., Pang G.-F., Li Y.-X. (2021). Imidacloprid increases intestinal permeability by disrupting tight junctions. Ecotoxicol. Environ. Saf..

[52] He L.-X., Wang J.-B., Sun B., Zhao J., Li L., Xu T., Li H., Sun J.-Q., Ren J., Liu R. (2017). Suppression of TNF-α and free radicals reduces systematic inflammatory and metabolic disorders: Radioprotective effects of ginseng oligopeptides on intestinal barrier function and antioxidant defense. J. Nutr. Biochem..

[53] Lätzer J., Papoian G.A., Prentiss M.C., Komives E.A., Wolynes P.G. (2007). Induced Fit, Folding, and Recognition of the NF-κB-Nuclear Localization Signals by IκBα and IκBβ. J. Mol. Biol..

[54] Malek S., Chen Y., Huxford T., Ghosh G. (2001). IκBβ, but Not IκBα, Functions as a Classical Cytoplasmic Inhibitor of NF-κB Dimers by Masking Both NF-κB Nuclear Localization Sequences in Resting Cells. J. Biol. Chem..

[55] Lindgren H., Olsson A.R., Pero R.W., Leanderson T. (2003). Differential usage of IκBα and IκBβ in regulation of apoptosis versus gene expression. Biochem. Biophys. Res. Commun..

[56] Wang W., Wu H., Yu H., Zhang X., Cui G., Wang K., Mao S., Pan Y. (2017). *Typhonium giganteum* Lectin Exerts A Pro-Inflammatory Effect on RAW 264.7 via ROS and The NF-κB Signaling Pathway. Toxins.

[57] Qin Y., Hua M., Duan Y., Gao Y., Shao X., Wang H., Tao T., Shen A., Cheng C. (2012). TNF-α expression in Schwann cells is induced by LPS and NF-κB-dependent pathways. Neurochem. Res..

[58] Liu Y., Lin X., Hao Z., Yu M., Tang Y., Teng X., Sun W., Kang L. (2023). Cadmium exposure caused cardiotoxicity in common carps (*Cyprinus carpio* L.): miR-9-5p, oxidative stress, energetic impairment, mitochondrial division/fusion imbalance, inflammation, and autophagy. Fish Shellfish Immunol..

[59] Xie W., Huang W., Cai S., Chen H., Fu W., Chen Z., Liu Y. (2021). NF-κB/IκBα signaling pathways are essential for resistance to heat stress-induced ROS production in pulmonary microvascular endothelial cells. Mol. Med. Rep..

[60] Cui J., Qiu M., Liu Y., Liu Y., Tang Y., Teng X., Li S. (2023). Nano-selenium protects grass carp hepatocytes against 4-tert-butylphenol-induced mitochondrial apoptosis and necroptosis via suppressing ROS-PARP1 axis. Fish Shellfish Immunol..

[61] Beltrán-Ortiz C., Peralta T., Ramos V., Durán M., Behrens C., Maureira D., Guzmán M.A., Bastias C., Ferrer P. (2020). Standardization of a colorimetric technique for determination of enzymatic activity of diamine oxidase (DAO) and its application in patients with clinical diagnosis of histamine intolerance. World Allergy Organ. J..

[62] Miao Z., Miao Z., Teng X., Xu S. (2022). Melatonin alleviates lead-induced intestinal epithelial cell pyroptosis in the common carps (*Cyprinus carpio*) via miR-17-5p/TXNIP axis. Fish Shellfish Immunol..

[63] Chopyk D.M., Stuart J.D., Zimmerman M.G., Wen J., Gumber S., Suthar M.S., Thapa M., Czaja M.J., Grakoui A. (2019). Acetaminophen Intoxication Rapidly Induces Apoptosis of Intestinal Crypt Stem Cells and Enhances Intestinal Permeability. Hepatol. Commun..

[64] Venkateswaran K., Shrivastava A., Agrawala P.K., Prasad A.K., Devi S.C., Manda K., Parmar V.S., Dwarakanath B.S. (2019). Mitigation of radiation-induced gastro-intestinal injury by the polyphenolic acetate 7,8-diacetoxy-4-methylthiocoumarin in mice. Sci. Rep..

[65] Woznicki J.A., Flood P., Bustamante-Garrido M., Stamou P., Moloney G., Fanning A., Zulquernain S.A., McCarthy J., Shanahan F., Melgar S. (2020). Human BCL-G regulates secretion of inflammatory chemokines but is dispensable for induction of apoptosis by IFN-γ and TNF-α in intestinal epithelial cells. Cell Death Dis..

[66] Huang Y., Mo S., Jin Y., Zheng Z., Wang H., Wu S., Ren Z., Wu J. (2022). Ammonia-induced excess ROS causes impairment and apoptosis in porcine IPEC-J2 intestinal epithelial cells. Ecotoxicol. Environ. Saf..

[67] Qian Y., Shi C., Cheng C., Liao D., Liu J., Chen G.-T. (2023). Ginger polysaccharide UGP1 suppressed human colon cancer growth via p53, Bax/Bcl-2, caspase-3 pathways and immunomodulation. Food Sci. Hum. Wellness.

[68] Ke N., Godzik A., Reed J.C. (2001). Bcl-B, a Novel Bcl-2 Family Member That Differentially Binds and Regulates Bax and Bak. J. Biol. Chem..

[69] Fu Y.-P., Yuan H., Xu Y., Liu R.-M., Luo Y., Xiao J.-H. (2022). Protective effects of *Ligularia fischeri* root extracts against ulcerative colitis in mice through activation of Bcl-2/Bax signalings. Phytomedicine.

[70] Pessoa J. (2021). Live-cell visualization of cytochrome c: A tool to explore apoptosis. Biochem. Soc. Trans..

[71] Gómez-Crisóstomo N.P., López-Marure R., Zapata E., Zazueta C., Martínez-Abundis E. (2013). Bax induces cytochrome c release by multiple mechanisms in mitochondria from MCF7 cells. J. Bioenerg. Biomembr..

[72] Jürgensmeier J.M., Xie Z., Deveraux Q., Ellerby L., Bredesen D., Reed J.C. (1998). Bax directly induces release of cytochrome c from isolated mitochondria. Proc. Natl. Acad. Sci. USA.

